# 

**Published:** 1866-12

**Authors:** 


### The Chicago Medical Journal.

**E. L. HOLMES, M.D., H. M. LYMAN, M.D., R. M. LACKEY, M.D. . . . Editors.**

All letters for the JOURNAL should be addressed to R. M. LACKEY, M.D., P. O. Box 2175, Chicago, Ill. *Office—169 South Dearborn Street.*

**TERMS—$2 PER ANNUM, IN ADVANCE.**

$3 if not paid before 1st of July of each year. Single Copies, 25 cents.

Clinical Instruction in Chicago .......... 583

### INDEX

### TO VOL. XXIII OF THE CHICAGO MEDICAL JOURNAL.

PAGE.

Abdomen, incised wound of....243
" and intestines, penetrating wound of....396
Affections of the nervous system, a lecture by C. E. Brown-Sequard, M.D.405
American Medical Association.... 89, 259, 307
" Ophthalmological Society, the annual meeting....366
Amputation of both legs, by G. K. Amerman, M.D.... 356
" of the hand and foot, by R. G. Bogue, M.D....360
Anasarca, puerperal....248
Anæmia, cerebro-spinal....555
Anæsthesia....83, 232
Anæsthetics, a new agent....476, 521
Aneurism of the aorta....281
" " abdominal aorta....460
" traumatic, Dr. R. N. Isham's case....222
" of the sciatic artery, treated by injection of perchloride of iron. 34
Anchylosis of the knee-joint....395
Arkansas, the hot springs of.... 4
Armenian cement....473
Asthenopia....145
Astigmatism....205
Atomizing tubes....520
Atropine upon the conjunctiva, poisonous effects of....403
Atropia and morphia, their antagonism....237

Belladonna, endermic poisoning by....286
Brainard, Prof. Daniel, the death of....529
Bromide of potassium in sleeplessness consequent upon uterine irritation..474
Bromine, an antidote to zymotic poison.... 18
Bronchocele, successful removal of, by W. Warren Greene, M.D....576

Calculus, urinary....280
California wines....489
Cancer, treatment with lemon-juice.... 36
" of the skin....401
Cattle plague, homœopathic treatment of.... 80
Caustic paste, Canquoin's.... 87

Cement, armenian....473
Cerebro-spinal meningitis....279
" anæmia, a case of....555
Codeia....191
Concussion, followed by jaundice.... 520
Conjunctivitis....477
Cook County Hospital....91, 429, 450
Correspondence from New York.... 11
County Hospital Clinics....163, 360, 398, 514
Chicago Charitable Eye and Ear Infirmary, sixth and seventh annual report....46, 379
Chicago Medical College....141
" Medical Society, proceedings of, .117, 170, 226, 279, 369, 401, 475, 520, 566
Chloroform, internal use of....522
Cholera, its portability.... 25
" epidemic at the County Hospital....450
" on Blackwell's Island....463
" its prevention in New York....465
" pathology and treatment....152, 176, 207, 351
" infantum, treatment with opium....475
Cirrhosis with tubercular peritonitis....567
Choroidal tumor....228
Clinical lectures on diseases of the eye, by E. L. Holmes, M.D., 1, 62, 104, 145, 202, 443, 507, 550
Clinical instruction in Chicago....583
Clinics, surgical, by Prof. E. Powell, M.D....14, 254
Clitoris, elephantiasis of....228
Cystic tumor of the ovary....569

Decision against river pollution....474
Death of Dr. Brainard....529
Dewitt County Medical Society....231
Diarrhœa, remedies for....466
Dyspnœa simulating croup....115

Early viability of the fœtus....414
Elephantiasis of the clitoris....228
Endoscopy....126
Epidemic cholera at the County Hospital....450
Erysipelas, a new remedy for....459
Excision of a portion of the humerus, by G. K. Amerman, M.D....358
" of a portion of the shaft of the humerus subsequent to excision of the head of the bone, by A. J. Baxter, M.D....388
Eye, clinical lectures on diseases of....1, 62, 104, 145, 202, 443, 507, 550
" and Ear Infirmary, sixth and seventh annual reports.... 46
" report on the diseases of, by E. L. Holmes, M.D....296

Fatty degeneration of the kidney, cases of....280

Fistula in ano........................................................................ 17
" " an operation for, two hundred years ago........................... 22
Fœtus, a case of early viability..............................................414
" punctured wound of...........................................................522
Fracture of the base of the skull..............................................229
Freer, Prof. J. W., introductory address by..................................491

Gangrene of the leg, from obstruction of the femoral artery, necessitating amputation..........................................................354
Gonorrhœa caused by Leucorrhœa ............................................391

Hard cataract...........................................................................281
Health bill of New York...........................................................181
Heart and kidneys, disease of..................................................567
Hematuria in an infant.............................................................114
Hernia, treated by inflation of the bowels................................. 18
Herrick, Prof. W. B., obituary notice of.................................... 93
Homœopathy, Dr. Kidd's........................................................... 81
Hog cholera in Ohio.................................................................572
Hospital for women and children.............................................487
Hourglass contraction before delivery.......................................151
Human deterioration................................................................ 35
Hypophosphites, their therapeutic value................................... 29

Iliac artery, ligature of the primitive........................................222
Illinois State Medical Society.............................................90, 324
Imperforate anus, case of..........................................................120
Intermittent fever, on the cause of............................................ 97
Intramural interments...............................................................188
Iowa State Medical Society........................................................317

Lemon juice in the treatment of cancer...................................... 36
Ligature of the primitive iliac artery..........................................222
" " right common iliac for aneurism of the abdominal aorta...460
Literary men and doctors.......................................................... 34
Lithectasis, a case of, in a girl three years of age.......................398
Local anæsthesia, method of producing.....................................232
" " cases of.....................................................................513
Localized bodily movements in the treatment of tuberculosis........539
Locomotor ataxy.........................................................283, 289, 294
Lymphatic glands, enlargement about the trachea, obstructing respiration.........................................................................564
Lynn, Dr. I. P., death of ...........................................................143

Medical intelligence, items of....................................................238
Meek, Dr. E. G., obituary notice of............................................527
Memorandum for those desirous of entering the Army Medical Corps.......430
Menstruation by a child, five years of age..................................387
Military Tract Medical Association.............................................375
Monstrosity, a remarkable case, in an adult................................ 80

Morgan County Medical Society....................................................374, 570
Morphia and atropia, antagonism of..................................................237
Morphine, a case of poisoning with...................................................559
Mortality reports of Chicago..........................................526, 527, 585
" of first labors....................................................................565
Mott, Valentine, last illness of.........................................................122
Mydriasis..............................................................................202
Myopia.................................................................................62
Myosis..................................................................................203

Narceine, physiological action of.......................................................473
Necrosis of the tibia....................................................................566
New apparatus..........................................................................369
Nitro-glycerine.........................................................................284
Nux vomica..............................................................................74

Obituary notice of Prof. W. B. Herrick...................................................93
" Dr. I. P. Lynn........................................................................143
" Dr. E. G. Meek........................................................................527
Observations on the "clamp" in the treatment of hemorrhoids, etc..........560
Obstetrics, a unique case in.............................................................67
Ophthalmoscope, as a means of investigating the diseases of the nervous system..........184
Opium in the treatment of cholera infantum.............................................475
Order of the police, giving physicians precedence at the bridges.................489
Osteo-myelitis.................................................................280, 363
Ovary, cystic tumor of..................................................................569

Paralysis of the muscles of the eye.....................................................550
Pathological Society of New York.......................................................255
Placenta prævia, cases of.......................................................247, 502
Pneumonia, the respiratory function in.................................................109
Poisoning with corrosive sublimate, a case of..........................................595
" laudanum, a case of...................................................................394
" morphine..............................................................................559
Pott's disease of the spine, diagnosis and treatment of...............................342
Premature delivery, on the propriety of inducing........................................71
Progressive locomotor ataxy..................................283, 289, 294, 390
Prolapsus of the rectum, treated with the clamp.........................................560
Puerperal anasarca......................................................................248
Pyelitis resulting from peritonitis.....................................................568

Rattlesnake bite, a case of.............................................................399
Regeneration of bone.....................................................................36
Reid's case of placenta prævia..........................................................502
Report on diseases of the eye, by E. L. Holmes, M. D...................................296
Respiratory function in pneumonia.......................................................109
Retention of the catamenia from obstruction.................................252, 369
" placenta, cases of.....................................................................241
Rock River Union Medical Society........................................................120

Rush Medical College....87, 491, 584

Sanitary Commission, contributions for the volumes of reports....472
Sanitary reports by N. S. Davis, M. D....402, 475, 570
Singular cause of death....569
Spinal meningitis....:241
Splinter imbedded for seven years in the muscles of the forearm....376
Stone in the bladder, two cases of....14, 16
Strabismus....507
Strangulated hernia, treated successfully by inflation of the bowels.... 18
Suppression of urine in cholera....520
Surgical report of the U. S. army.... 20
Sympathy between the auditory canal and the larynx....471
Syphilis....230
Syphilitic diseases, cases of....566

Talipes, the gutta-percha shoe in the treatment of....179
Thermometer, the use of, in clinical medicine....193
Thoracentesis, two cases of, by J. W. Freer, M.D....385
Trichina....36, 230
" spiralis—poisoning by eating trichinous pork....337
Tumor on the left side of the neck.... 14
Tumors removed from the larynx in a case of aphonia.... 70
Twins with one placenta, two cords and one set of membranes....449
Typhoid fever....521

Ulcers, treatment of chronic....232
Urethra, stricture of....514
" perineal section of, by G. K. Amerman, M. D....517
Urinary calculus....280

Valedictory address to the graduating class of Rush Medical College, by Prof. Allen.... 49
Valentine Mott, last illness of....122
Ventilation of sewers.... 35
Vivisections....573

